# Takayasu's arteritis presenting with temporary loss of vision in a 23-year-old woman with beta thalassemia trait: a case report

**DOI:** 10.1186/1752-1947-5-466

**Published:** 2011-09-20

**Authors:** Mohammad G Ishaq, Fahad A Shabbir

**Affiliations:** 1 Department of Medicine, Jinnah Medical College Hospital, SR-6 Sector 7/A KIA, Korangi, Karachi, Pakistan

## Abstract

**Introduction:**

The simultaneous presence of Takayasu's arteritis and beta thalassemia trait is a rare combination. To the best of our knowledge, this is the first case report on Takayasu's arteritis and beta thalassemia presenting together.

**Case presentation:**

This is a case report of a 23-year-old Asian woman of Pakistani descent who presented with a headache, blurred vision and dizziness.

**Conclusion:**

The correct diagnosis of our patient was based on clinical suspicion, appropriate imaging studies, and deliberation of the differential diagnosis. The management of our patient depended on the correct diagnosis of both the diseases.

## Introduction

Takayasu's arteritis (TA) is an autoimmune, chronic, progressive, large-vessel vasculitis that usually affects young adults, especially women. The disease can affect all races and ethnic groups. The diffuse nature of this vasculitis can involve multiple organ systems to varying degrees and can present with a wide range of symptoms [1], with an incidence of one to two cases per million people per year [2]. Beta thalassemia trait is an autosomal recessive disorder characterized by a point mutation on the beta-globin chain gene on chromosome 11, resulting in the defective synthesis of the beta-globin chain of hemoglobin [3]. To the best of our knowledge, the incidence of TA with beta thalassemia trait has not previously been reported in the literature.

### Case Presentation

A 23-year-old Asian woman of Pakistani descent presented with blackouts, blurring of vision and headache for more than two months duration. The headaches started in the frontal region then radiated to the whole head, were moderate in intensity and were associated with vertigo, dizziness, palpitations and postural weakness. Her past medical history revealed that she had been diagnosed with epilepsy two months previously, after which she had been given antiepileptic medication. She had been using the medication regularly since being diagnosed. On general examination, pulses in both her upper limbs were deficient and so her blood pressure could not be measured. Our patient was also found to be anemic. On cardiovascular examination a bruit was heard over her left subclavian fossa. Fundoscopy revealed optic disk cupping with irregular margins in her right eye; her left eye was unaffected. All other examination, including respiratory and central nervous system examinations, were unremarkable. A psychiatric evaluation was also inconclusive.

Our patient had anemia (hemoglobin 10 g/dL), thrombocytosis (494,000/mL) and raised erythrocyte sedimentation rate (ESR) (35 mm/hr). The morphology of the red blood cells showed microcytosis and hypochromasia. An investigation into the serum ferritin revealed that it was well above the normal range (315.5 ng/mL). Hemoglobin electrophoresis presented with a mean corpuscular volume of 58.7 fL, mean corpuscular hemoglobin of 19.2 pg, and hemoglobin A2 of 4.7%. Liver enzymes were significantly raised (direct bilirubin 0.3 mg/dL, alanine transaminase 152 U/L, alkaline phosphatase 317 U/L). The C-reactive protein test was also reactive. In addition the following investigations were unremarkable: serum iron, total iron binding capacity and transferrin, antinuclear antibodies, electrocardiogram, echocardiograph and electroencephalogram (EEG).

**Figure 1:**
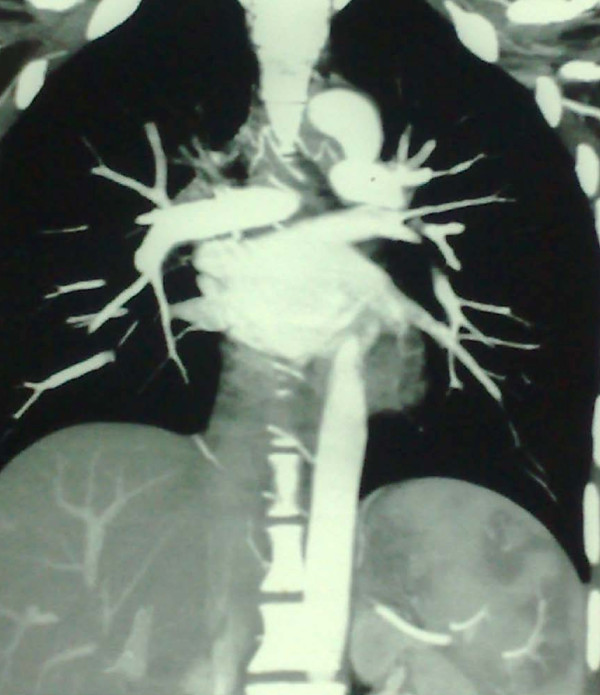
**CT-A of arch of the aorta and its major branches showing diffuse intimal thickening, with narrowing of the origins of major branches of the arch of the aorta involving brachiocephalic, right common carotid and left subclavian arteries**.

A computed tomography angiogram (CT-A) of her chest showed a normal ascending aorta, descending aorta and arch of the aorta but there was diffuse intimal thickening of major branches of the aorta including the brachiocephalic, right common carotid, and left subclavian arteries (Figure 1). There was extensive collateral circulation in the subcutaneous tissue in her anterior and posterior neck, chest and axillae.

Our patient had been taking antiepileptic medication for over a month. This was immediately halted because her liver enzymes were elevated above normal range; this decision was also supported by the negative results of the EEG report (the alpha wave was present on closure of eye and had a frequency of 10 cycles per second; which disappeared when the patient was instructed to open her eyes, beta, theta and delta waves had frequencies of 14, 5 and 3 cycles per second). Our patient was kept on one milligram per kilogram bodyweight per day of corticosteroid and was kept under a weekly follow-up for two months to monitor her response to the treatment. Our patient is responding well.

## Discussion

Based on the clinical history, examination, imaging studies and serum electrophoresis our patient was diagnosed with Type I TA with beta thalassemia trait. TA may present with nonspecific symptoms such as fever, arthralgia and weight loss. It may also present with systemic complaints depending on the site of involvement; for example neurological symptoms like dizziness (33%) and impairment of vision (20-30%), carotid bruit (70%), deficit of upper limbs pulses (53%) and the deficit of other pulses, all of which were seen on examination of our patient [4]. Patients with TA may also present with epilepsy [5]. Fifty percent of patients with TA have a normal ESR [6]. Patients with beta thalassemia trait also have a raised ESR. Anemia in TA can be normocytic and normochromic, but our patient presented with microcytic hypochromic anemia which is commonly due to an iron deficiency in females of reproductive age in this part of the world [7]. To further confirm our diagnosis of beta thalassemia, a serum electrophoresis was done to firmly establish that the anemia was due to beta thalassemia trait and to exclude iron deficiency anemia.

The differential diagnosis of TA may include congenital tissue matrix disorders (Marfan and Ehler-Danlos syndromes), infectious large vessel aneurysm (mycobacterial, syphilitic, or fungal) and autoimmune diseases (systemic lupus, Cogan syndrome and Behçet's disease); however these disorders were ruled out because they are not associated with large vessel stenosis as seen in TA. Large vessel vasculitis was ruled out because it is associated with the advanced age (Kawasaki disease and giant-cell arteritis). Sarcoidosis is also considered as a differential of TA, but is only diagnosed after all the differentials have been ruled out.

Current studies postulate that non-invasive imaging techniques are very helpful in the early diagnosis of TA, such as magnetic resonance imaging, ultrasound and 18F-fluorodeoxyglucose positron emission tomography. In comparison to standard angiography, they also provide a tool for a comprehensive monitoring of the disease [8]. Treatment of TA is a real challenge for the clinicians and in spite of current treatment vascular lesions develop frequently. Remission occurs in patients using immunosuppressive drugs (for example methotrexate) along with corticosteroids, but unfortunately reactivation is common when the dose of corticosteroids is brought down. The use of surgical and endovascular procedures are safe and associated with low mortality and morbidity. Good results have been achieved using bypass grafts, whereas percutaneous transluminal angioplasty gives better results for smaller lesions. Conventional stents used in vessel patency are just not good enough in the long-term [9]. The use of tumor necrosis factoralpha inhibitors and drug-eluting arterial stents has been shown to improve prognosis in severe disease. Initial results show promising outcomes and higher chances of therapeutic success may be attained by newer drugs like rapamycin (sirolimus) and everolimus. These drugs inhibit myointimal proliferation thereby effectively inhibiting vessel intimal hyperplasia and inducing expression of heme oxygenase-1 [10].

Beta thalassemia trait is not treated actively; erythropoesis is ineffective and down-regulation of iron absorption is also altered. Thus occurs because of an excess of the growth differentiation factor 15 (GDF-15), which is produced by the erythroid tissue of the bone marrow. GDF-15 inhibits hepcidin gene expression, which in turn increases iron absorption from the gastrointestinal tract [11]. Hence the use of oral iron supplementation should be monitored by measuring the serum ferritin levels at regular intervals. Beta thalassemia trait is not a life-threatening condition but can affect the quality of life due to mild or moderate anemia and should only be treated if the patient becomes symptomatic. The coexistence of other disease with beta thalassemia trait has been shown in studies including asthma [12] and mood disorders [13]. Genetic counseling is an essential part of managing such patients in our setting, where beta thalassemia trait testing is not done routinely.

## Conclusion

The simultaneous occurrence of TA and beta thalassemia trait is a rare incidence which has not previously been reported; therefore it is important that such a case be presented in the international literature as a reference point. Clinical suspicion, appropriate imaging, and the consideration of the differential diagnosis are important for the correct diagnosis and management of patients with TA and beta thalassemia trait.

## Abbreviations

CT-A: computed tomography angiogram; EEG: electroencephalogram; ESR: erythrocyte sedimentation rate; GDF-15: growth differentiation factor-15; TA: Takayasu's arteritis.

## Consent

Written informed consent was obtained from the patient for publication of this case report and any accompanying images. A copy of the written consent is available for review by the Editor-in-Chief of this journal.

## Competing interests

The authors declare that they have no competing interests.

## Authors' contributions

MI was responsible for complete diagnosis, treatment and narrative of the patient's history. FS conducted the examination, wrote the manuscript and is responsible for the correspondence. All authors read and approved the final manuscript.
